# Evaluation of the Potential of Sewage Sludge Mycobiome to Degrade High Diclofenac and Bisphenol-A Concentrations

**DOI:** 10.3390/toxics9060115

**Published:** 2021-05-23

**Authors:** Ulises Conejo-Saucedo, Alejandro Ledezma-Villanueva, Gabriela Ángeles de Paz, Mario Herrero-Cervera, Concepción Calvo, Elisabet Aranda

**Affiliations:** 1 Institute of Water Research, University of Granada, Ramón y Cajal, 4, Fray Luis Bldg, 18071 Granada, Spain; uconejo@correo.ugr.es (U.C.-S.); ledezmavilla@gmail.com (A.L.-V.); gangeles@correo.ugr.es (G.Á.d.P.); herrero_marcer@externos.gva.es (M.H.-C.); ccalvo@ugr.es (C.C.); 2 Department of Microbiology, University of Granada, 18071 Granada, Spain

**Keywords:** sewage sludge, pharmaceuticals active compounds, bioremediation, endocrine disruptors, shotgun-sequencing technologies, fungi, bacteria

## Abstract

One of the most challenging environmental threats of the last two decades is the effects of emerging pollutants (EPs) such as pharmaceutical compounds or industrial additives. Diclofenac and bisphenol A have regularly been found in wastewater treatment plants, and in soils and water bodies because of their extensive usage and their recalcitrant nature. Due to the fact of this adversity, fungal communities play an important role in being able to safely degrade EPs. In this work, we obtained a sewage sludge sample to study both the culturable and non-culturable microorganisms through DNA extraction and massive sequencing using Illumina MiSeq techniques, with the goal of finding degraders adapted to polluted environments. Afterward, degradation experiments on diclofenac and bisphenol A were performed with the best fungal degraders. The analysis of bacterial diversity showed that Dethiosulfovibrionaceae, Comamonadaceae, and Isosphaeraceae were the most abundant families. A predominance of Ascomycota fungi in the culturable and non-culturable population was also detected. Species such as *Talaromyces gossypii*, *Syncephalastrum monosporum*, *Aspergillus tabacinus*, and *Talaromyces verruculosus* had remarkable degradation rates, up to 80% of diclofenac and bisphenol A was fully degraded. These results highlight the importance of characterizing autochthonous microorganisms and the possibility of selecting native fungal microorganisms to develop tailored biotransformation technologies for EPs.

## 1. Introduction

Emerging pollutants (EPs), such as pharmaceutical active compounds, endocrine-disrupting compounds, personal care products, fragrances, detergents, and disinfectants, have an increasing presence in the environment, particularly in aquatic ecosystems [1,2]. These emerging and unregulated contaminants end up in the soils and water bodies, causing an imbalance in the microbial communities, which culminate in the possible development of resistance to antimicrobial agents and, finally, endanger human health and environmental stability. The occurrence of EPs has been observed to range from nanograms per liter to micrograms per liter in aquatic environments. This seems to be a direct consequence of discharges of raw or treated wastewater (from hospital, municipal or industrial origin) after their treatment in wastewater treatment plants (WWTPs), and the use of sewage sludge as organic soil amendments [3,4,5,6,7]. Several aspects, such as out-of-date installations, a high load of these compounds in the influent, and the usual persistence in the environment of these molecules that bind to organic matter and clay particles, have led towards the proposal of alternatives that support this notorious breach in the environmental management of EPs [8]. In recent studies, approximately 60 EPs have been detected in WWTPs (influent, treated effluent, sewage sludge, and biosolids) including diclofenac (DFC) and bisphenol A (BPA) [9,10].

Diclofenac ([2- (2,6-dichloroanilino) phenyl] acetic acid) (diclofenac; DFC) is one of the most frequently, non-steroidal anti-inflammatory drugs, detected in the environment, and the most used constituent in drug formulations [11]. On the other hand, bisphenol A (2,2-Bis[4-hydroxyphenyl]propane) (BPA) is a synthetic chemical widely used as a material for the synthesis of epoxy resins and polycarbonate plastics, among other applications. BPA is considered a toxic compound and it has been identified as a chemical endocrine disruptor, which affects both animals and humans, producing adverse effects on development, reproduction, cardiovascular changes, and negative effects on the immune and the neurological systems [12,13]. 

As a result of their inappropriate usage and disposal, DFC and BPA are introduced into the environment through different routes, including industrial or domestic effluents, industrial or domestic solid waste, medical waste from healthcare, veterinary facilities, landfill leachate, biosolids, wastewater effluent, and treated sewage sludge [14,15]. In these last two instances, it has been observed that DFC and BPA become restrained in a complex matrix and organic matter, making it even more difficult for the typical microbial treatment of the WWTPs [2]. According to a study performed by Quintana et al. [16], the average removal of diclofenac in a WWTP was one of the lowest of the EPs in activated sludge. Yu et al. [17] also reported low degradation rates of DFC (30%) over 50 days. 

There are different approaches for the removal and degradation of DFC and BPA by physical and chemical methods under laboratory conditions [18,19,20], and using wastewater in combination with agri-food waste types [21]. However, some of these methods are not effective, or they generate intermediary substances that are even more toxic than the original [22].

Recent advances in the study of microbial communities through next-generation sequencing (ARNr 16S-ITS markers) have contributed to a better understanding of the microbial diversity in sewage sludge coming from WWTPs. These options allow for the interpretation of the microcosm abundance or biological interactions among species in order to enhance suitable bioaugmentation processes aimed at prompting the degradation of xenobiotic compounds such as DFC [23,24,25]. 

Filamentous fungi play an important role in the degradation of EPs, since they have a series of physical, chemical, and molecular characteristics that make them versatile organisms with a great potential for bioremediation. They can form a large proportion of biomass and biofilms, and they can obtain their source of energy from different origins by a great variety of hydrolytic enzymes. In addition, they can be adaptive to a wide range of temperatures and pH; they produce secondary metabolites that prevent the growth of other competitors, and the composition of the cell wall protects them from toxic compounds through the absorption process [26]. Fungal systems have been considered an effective solution for removing micropollutants, particularly those that inhabit polluted environments, and some approaches have been tested in wastewater treatments [27,28,29].

This has opened an opportunity for fungal species to propose a complementary treatment that grants economical and term advantages over the bacterial, chemical or physical treatment of pharmaceutical active compounds (PhACs) [30,31] and EPs [10,27,32]. 

The degradation potential of the EPs’ different filamentous fungi has previously been studied, for instance, DFC in studies with *Trametes versicolor*, *Trametes trogii*, *Aspergillus niger*, *Yarrowia lipolytica,* and *Phanerochaete chrysosporium*. *Trametes trogii* was found to be the most efficient strain, with a 100% DFC degradation rate in 48 h [33]. In addition, the fungus *Trametes versicolor* was able to remove, partially or completely, 46 of the 51 pharmaceutical active compounds detected in the wastewater from hospitals [30].

In a recent study, an isolated fungus (*Aspergillus luchuensis*) from municipal wastewater demonstrated the ability to grow in the presence of carbamazepine, diclofenac, ibuprofen, and sulfamethoxazole [34]. A high removal efficiency rate (>99.9%) of DFC was observed in synthetic wastewater medium after 10 days of incubation, while >98% of DFC was disposed of in non-sterile wastewater [34]; this demonstrated that the application of autochthonous or native fungi adapted to terrestrial or aquatic polluted habitats provides an important tool for the biodegradation or biotransformation of EPs [35,36,37].

In this research, we turned to the isolation of fungi from sewage sludge, and the study of their capability to transform DFC and BPA as representative compounds of EPs. Later, we studied the microbial community present in this sewage sludge (i.e., fungi and bacteria) to propose a bioaugmentation-based treatment for application in processes that involve sewage sludge management processes, such as a composting procedure that promotes the growth of autochthonous microbial species in order to take advantage of their morphological, physiological, and genetic features, leading to a bigger intake of carbon sources.

## 2. Materials and Methods

### 2.1. Solutions and Reagents

Diclofenac sodium salt (CAS: 15307-79-6) (≥98%) and bisphenol A (CAS 80-05-7) (≥97%) 4,4′-(propane-2,2-diyl) diphenol were purchased from Sigma-Aldrich (St. Louis, MO, USA) and Acros Organics (Geel, Bélgica), respectively. Acetonitrile and water for high-performance liquid chromatography (HPLC) analysis were acquired from Panreac Química SLU ITWReagents^®^ (Barcelona, Spain) and BDH Prolabo^®^ (Johannesburg, South Africa), respectively. DFC and BPA stock solutions were prepared at 5 mM and later sterilized by filtration (0.22 µm). The stock solution of DFC and BPA was used at a final concentration of 100 µM in the bioremediation experiments at flask scale.

### 2.2. Sampling

Sewage sludge composite samples were collected from the WWTPs located in Vegas del Genil, Granada, Spain (37.1912150901147, –3.6760580525127673) following biosafety protocols and deposited in 1 L sterile amber glass containers with Teflon-lined lids. Three different samples were taken from the site (LM, L1, and L2). Samples were transported at 4 °C. Part of the sample was used for fungal isolation, and the rest was frozen at –20 °C for total DNA extraction. 

### 2.3. Fungal Strain Isolation and Spore Collection 

The strains were isolated according to the methodology described by Wasksman [38]. One gram of sludge was added and mixed with 9 mL sterile saline solution (0.9% NaCl) to form an aliquot. Dilutions (1 x 10^-3^) were used and inoculated in MEA (malt extract agar) medium plates with tetracycline and streptomycin as antibiotics (50 and 25 μg mL^−1^, respectively). MEA plates were incubated at 28 °C for 5 days. Distinct morphological colonies were selected and isolated from the diluted solutions growing on the mentioned plates and then sub-cultured on MEA medium at 28 °C for 7 days. Culture samples were stored at 4 °C and –70 °C in glycerol (15%).

### 2.4. DNA Extraction 

Fresh, pure cultures of isolated fungi were processed to extract genomic DNA using the PrepMan^®^Ultra kit that applies a heating-based lysing procedure. Consequently, a fresh colony was resuspended in 100 μL of lysing reactive and heated to 100 °C for 10 min, followed by a cool down at room temperature for 2 min. Afterwards, samples were centrifuged at 13.680× *g* for another 2 min, and, finally, 50 μL of the supernatant was transferred into a new tube to be stored at –20 °C. The DNA for sewage sludge was obtained using the MoBio PowerSoil^®^ DNA isolation kit (Palex Medica, SA, Sant Cugat del Valles, Barcelona, Spain) following the manufacturer’s instructions, which included the application of a mechanical lysis and isolation procedure [39].

### 2.5. PCR Identification 

Genomic DNA obtained from pure fungi cultures were amplified with the oligonucleotides ITS1 (TCCGTAGGTGAACCTGCGG) and ITS4 (TCCTCCGCTTATTGATATGC) in a thermocycler (GeneAmp PCR system 2700/Applied Biosystems). For PCR amplification, the DreamTaq DNA polymerase kit (Thermo Scientific) was used, and a final reaction volume of 50 µL and 20 pmol of each primer and 1 µL of DNA were added. PCR programming conditions were 90 °C—30 s, 55 °C—30 s, 72 °C—60 s, and 72 °C—600 s for 35 cycles. Amplicons were run in agarose gel 1% (w/v) with TBE 1x buffer to later be excised and purified with NucleoSpin^®^ gel and PCR cleanup kit (Macherey–Nagel). DNA sequence analyses were carried out in a genetic analyzer (ABI PRISM 3130XL/Applied Biosystems, Foster City, CA, USA), using and following the manufacturer’s instructions for the BigDye Terminator v3.1 cycle sequencing kit (Applied Biosystems). The nucleotide sequences were submitted to the online BLAST search engine of the National Centre for Biotechnology Information (http://www.ncbi.nlm.nih.gov/BLAST).

### 2.6. Bacteria and Fungi Illumina Sequencing 

To determine bacterial and fungal diversity, sequencing libraries and core amplicon data analyses were generated using the Earth Microbiome Project protocols (www.earthmicrobiome.org) [40]. On the one hand, for bacteria, the hypervariable zone V4 of the 16S ribosomal DNA (16S) was amplified using 515 F and 806 R oligos [41]. The reaction was assembled for each oligo pair in triplicate using 1X Ex Taq buffer (Takara Bio Inc., Shinga, Japan). The mixture consisted of 515F/806 R pair oligos (150–200 nM), 1 μL of DMSO, 200 μM of dNTPs mixture (Takara Bio Inc., Shinga, Japan), 0.56 mg mL^–1^ BSA (Roche, Basel, Switzerland), 0.025 U μL^–1^ of TaKaRa Ex Taq (Takara Bio Inc., Shinga, Japan), and 4 ng of template DNA.

The thermocycler program for bacteria consisted of the following steps: 3 min at 95 °C for initial denaturalization, followed by 25 cycles of 20 s at 95 °C, 20 s at 60 °C, and 40 s at 72 °C, finishing with 2 min at 72 °C for the final extension. On the other hand, for fungi, the ITS region was amplified using ITS1–ITS2 primers [42]. The PCR program was performed as continued: 3 min at 95 °C for initial denaturalization followed by 30 cycles of 30 s at 95 °C, 30 s at 51 °C, and 30 s at 72 °C, finishing with 5 min at 72 °C for the final extension.

Sequence libraries were prepared with Golay Barcode reverse primers (12 base pairs) [43]. Sequencing was run using the Illumina MiSeq reagent v3 600 cycle and the Illumina-MiSeq platform at the Vincent J. Coates Genomics Sequencing Laboratory (UC Berkeley, Berkeley, UC, USA).

### 2.7. Data and Bioinformatics Analysis 

Bacterial and fungi raw sequences were analyzed using QIIME2 version 2020 [44]. Quality filtering (clustering and denoising) was executed using DADA2 [45]. Fungal analyses were performed using additional filtering based on the UNITE (v7.2) database to separate the operational taxonomic units (OTUs) classified to the kingdom fungi. The threshold of 99% 16S rRNA gene sequence identity was used for bacteria and a 97% threshold of the ITS2 gene sequence identity was used for fungi.

Illumina sequencing bar plots were generated using the plugin q2-diversity in QIIME2.

### 2.8. Biodegradation at Flask Scale

DFC and BPA degradation tests by the isolated fungal strains were carried out with sterile liquid kirk medium [46] (25 mL) added to 100 mL flasks and inoculated with 1 × 10^5^ spores/mL (final concentration). The spores were obtained using a standard protocol and counted using the hemocytometer method. Pellet formation was performed in shaking flasks at 120 rpm and cultured for 48 h at 28 °C. One hundred micromolar of DFC or BPA was added to each flask and subsequently incubated at 28 °C for 72 h. The following two different biodegradation systems were evaluated: (1) positive control (active biomass with the pollutant); (2) negative control (biomass inactivated by autoclave).

During the kinetics degradation, sacrificial flasks were taken every 6 h. Samples were collected from each flask to individually analyze pH, fungal biomass, and glucose content as described below. Later, the flasks were treated with 1.8 volumes of ethanol, followed by sonication (5 min on/1 min rest for 15 min) to release the DFC/BPA adhered to the glass and/or fungal biomass. The resulting solution was centrifuged and afterwards the residual DFC/BPA was analyzed by HPLC. 

The total sugar content of the culture media was evaluated using the DNS colorimetric method by Miller (1959) [47]. Biomass was quantified by the gravimetric method and pH using test strips 0–14 (Macherey–Nagel, Dueren, Germany).

All sampling in the different degradation systems were performed in triplicate.

### 2.9. Residual Diclofenac and Bisphenol A Analysis

Both compounds (DFC/BPA) were measured by HPLC employing an Agilent^®^ 1050 HPLC system (Waldbronn, Karlsruhe, Germany) equipped with a diode array detector (DAD; 190–700 nm) and a Synergi Fusion RP C18 column (4 μm, 4.6 Å~ 150 mm; Phenomenex^®^, Madrid, Spain). Ten microliters of each sample (DFC/BPA) was injected. For DFC analyses, an isocratic flow rate of 0.9 mL min^-1^ and a buffer acetonitrile/water ratio of 85:15 (containing H3PO4 0.01%) were used for elution. For BPA, an isocratic flow rate of 1 mL min^-1^ and a buffer acetonitrile/water ratio of 65:35 (containing H_3_PO_4_ 0.01%) were used. Peak areas of UV detection at 278 nm were interpolated into a generated standard curve using 10–100 μM. The removal rate was calculated according to Olicón-Hernandez et al. (2019) [31].

### 2.10. Enzymatic Analysis

Laccase activity (EC 1.10.3.2) was determined spectrophotometrically using ABTS (2,2′-azino-bis (3-ethylbenzothiazolin-6-sulfonic acid; 0.3 mM) at an absorbance of 420 nm in 100 mM sodium citrate buffer (pH 4.5) [48]. Manganese peroxidases (MnP) activity (EC 1.11.1.13) was also measured spectrophotometrically using sodium malonate buffer (100 mM, pH 4.5) at an absorbance of 270 nm for 30 seconds in the presence of H_2_O_2_ (0.1 mM) [49]. Unspecific peroxygenase (UPO) activity (EC 1.11.2.1) was determined through the oxidation of veratryl alcohol (5 mM) at an absorbance of 310 nm, using 100 mM of potassium phosphate buffer (pH 7) for 17 seconds and in the presence of H_2_O_2_ [50].

## 3. Results and Discussion

### 3.1. Fungal Culturable Population

In this study, a total of 17 isolates were isolated from three samples of sewage sludge, and molecular analyses were performed to identify the different isolates. The results showed that most of the isolates belonged to the phylum Ascomycota, *Aspergillus* being the most frequent genus identified (five isolates), while the second most frequently isolated were *Talaromyces* and *Galactomyces*, with two isolates each. The genera that presented only one isolate included *Syncephalastrum*, *Saccharomycetales* sp., *Byssochlamys*, *Trichoderma*, *Scedosporium*, *Sporothrix,* and *Mucor* (Table 1). Some of them have been previously described as potential degraders of PAHs [51].

It is well known that sewage sludge contains a variety of microorganisms, such as bacteria, fungi, and viruses. Microorganisms isolated in different samples from wastewater treatment studies showed that the fungal species were largely predominant due to the fact of their capability to adapt to the environmental and operational conditions. In addition, a great diversity of fungi was found in sewage sludge, and *Aspergillus*, *Trichoderma*, and *Penicillium* were commonly found genera in this matrix [28,29].

### 3.2. Bacterial Community Structure

**Figure 1:**
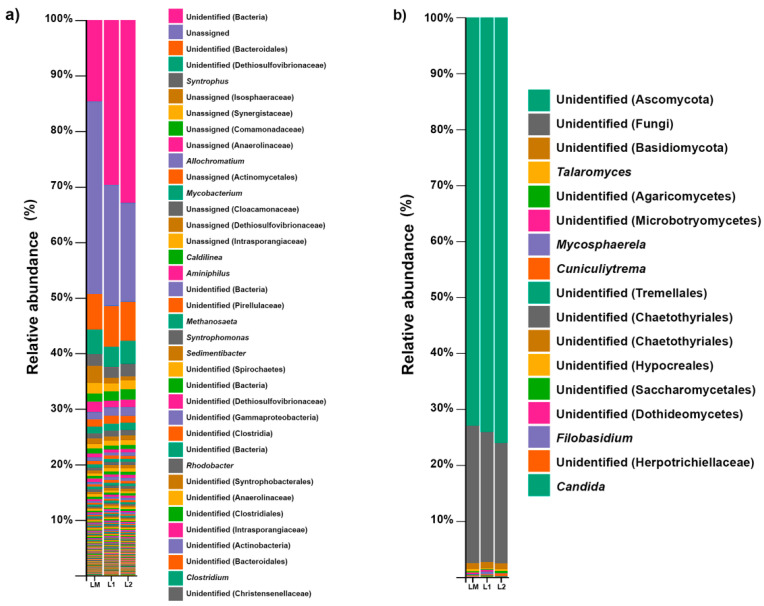
Percentage of relative abundance of the bacterial (**a**) and fungal (**b**) communities at the genus level, using Illumina MiSeq for the three samples. Sludge mix (LM), sludge sample 1 (L1), and sludge sample 2 (L2).

The bacterial community diversity at the genus level in the three samples (LM, L1, L2) is represented in Figure 1a. The sub-OTUs assignation in samples was examined using the 16S rRNA reference database (Greengenes) [52].

The unassigned bacteria domain (25.6%) and Bacteroidales order (6.9%) were the most abundant in sewage sludge. Moreover, the most abundant families were Dethiosulfovibrionaceae (4.1%), Comamonadaceae (1.6%), and Isosphaeraceae (1.5%).

The clustering of sequences in OTUS with a similarity percentage of 99% allowed for the detection of 136 genera, but only 7 had a relative abundance >0.5%. We detected *Syntrophus* (2.0%), *Allochromatium* (1.4%), *Mycobacterium* (1.2%), *Caldilinea* (0.7%), *Aminiphilus* (0.5%), *Syntrophomonas* (0.5%), and *Sedimentibacter* (0.5%) as the most abundant genera (Figure 1a). These genera include important degraders that could involve the possible natural degradation of xenobiotics. A recent metagenomic analysis from hydrothermal vent sediments demonstrated the presence of sulfate-reducing bacteria belonging to the Dethiosulfovibrionaceae family [53]; sulfate-reducing bacteria could be useful in bioremediation environments such acid mine drainage decontamination [54,55].

In the same way, they have an ecological significance in terms of the treatment of food industrial wastewater [56]. Otherwise, species of the genus *Syntrophus* (*S. aciditrophicus*) have shown that they are capable of degrading aromatic compounds, such as benzoate, cyclohexane carboxylate, and cyclohex-1-ene carboxylate, in co-culture with hydrogen using methanogens or sulfate reducers [57].

It is remarkable that part of the genera found in this study were bacteria well adapted to this microenvironment, such as the species of genus *Allochromatium*, which are capable of reducing thiosulfate to sulfur compounds as electron donors for growth [58]. The genus *Caldilinea* is capable of growing in environments with low concentrations of carbohydrates and oxygen and, at the same time, degrade biopolymers in environments such as activated sludge from WWTPs [59,60].

The genus *Mycobacterium* was frequently found to be present in global WWTPs, especially in municipal WWTPs [61]. Several *Mycobacterium* strains were human pathogenic species. However, some species, such *Mycobacterium austroafricanum* and *Mycobacterium sp*., have been identified to have the ability to degrade aromatic hydrocarbons [62,63,64].

We also detected members of the phyla Euryarchaeota (Archaea), including the genera *Methanosaeta*, *Methanobrevibacter*, *Methanosphaera*, *Methanobacterium*, *Methanospirillum*, *Methanolinea*, and *Methanomassiliicoccus*, which were detected at a <0.5% abundance. The rest of the bacterial community (relative abundance <0.5%) is represented in the Supplementary Materials Table S1.

### 3.3. Fungal Community Structure

Figure 1b shows the composition of the fungal diversity at the genus level in the three samples of sewage sludge (LM, L1, L2), based on the observed sub-OTUs of the ITS2 gene and mapped with the UNITE references database [65].

Ascomycota (74.6%), unidentified fungi (23%), and Basidiomycota (1%) were the most abundant phyla in the sewage sludge samples (Figure 1b). At the family level, Trichocomaceae and Mycosphaerellaceae were the most abundant families. The fungal community data show that the main genera were *Talaromyces*, *Mycosphaerella*, *Cuniculitrema,* and *Filobasidium* with >1% abundance. The fungal community structure shows the presence of the genus *Candida* (>1%). The species of these genera are considered opportunistic human pathogens. 

Many of the fungi belonging to the Ascomycota phylum play a crucial role in the degradation of a wide variety of xenobiotic compounds through oxidation processes. Recent studies show the presence and diversity of fungi adapted to these environments and their contribution to the degradation of these pollutants [66,67]. Additionally, Ascomycota predominantly represented the composition of the fungal community in hospital wastewaters [68]. This diversity of fungi is common in populations of wastewater treatment plants [69,70]. Several studies demonstrated the use of different species of the genera *Penicillium*, *Aspergillus,* and *Trichoderma* as a useful tool in different biological sludge treatments [29]. 

### 3.4. Diclofenac Degradation Experiments

Consumption of DFC by the 17 isolated fungal strains during 72 h in laboratory conditions was reported in only 6 of them, *Talaromyces gossypii*, *Syncephalastrum monosporum*, *Aspergillus tabacinus*, *Talaromyces verruculosus*, *Aspergillus terreus* and *Aspergillus cejpii*, as it is shown in Table 1. 

**Figure 2:**
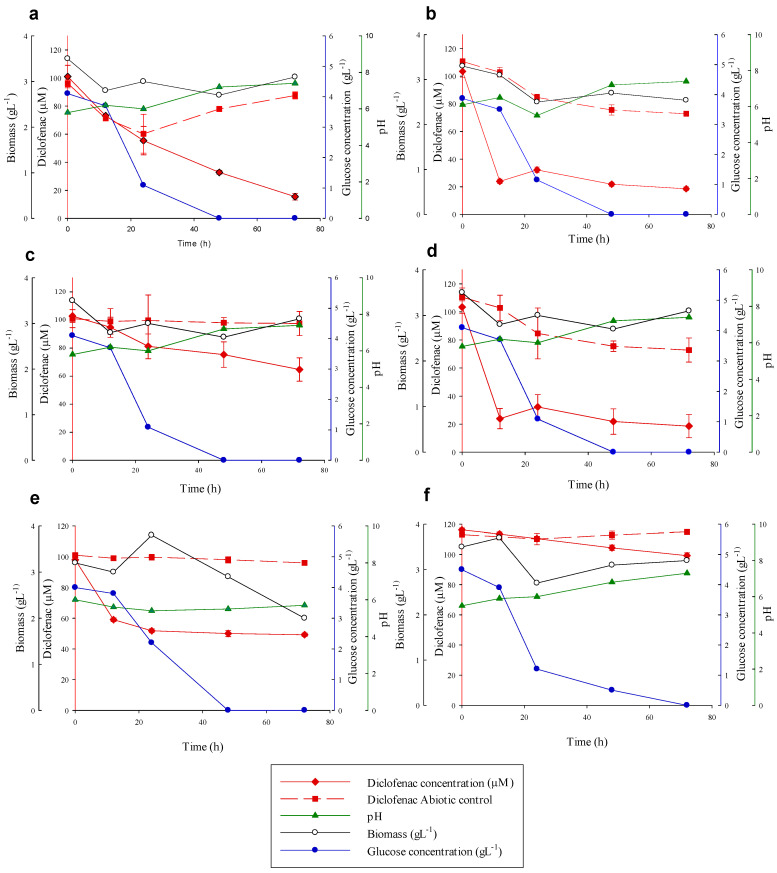
Comparison of diclofenac elimination by *Talaromyces gossypii* (**a**), *Syncephalastrum monosporum* (**b**), *Aspergillus tabacinus* (**c**), *Talaromyces verruculosus* (**d**), *Aspergillus terreus* (**e**), *Aspergillus cejpii* (**f**). Diclofenac concentration (red diamond), glucose concentration (blue circles), pH (green triangle), biomass (white circles) and the diclofenac abiotic control (red square) were measured. Error bars represent standard deviation of three independent replicates.

After the incubation period, *T. gossypii* showed the maximum DFC removal percentage with 84.6%, *S. monosporum* showed 82% and *A. tabacinus* showed 76% DFC removal. Additionally, *A. terreus, T. verruculosus,* and *A. cejpii* showed lower removal percentages of 49.7%, 37% and 14.6% of DFC, respectively (Figure 2). 

The ability to secrete extracellular enzymes, especially those related with lignin degradation, is a common feature for Basidiomycota fungi, and is also related with the presence of lignocellulosic substrates. Despite the fact that some Ascomycota are able to secrete ligninolytic enzymes, interestingly, in this experiment, no activity of extracellular enzymes (Lac, MnP, UPO) was detected (data not shown). Thus, the CYP450 enzyme probably could be involved in the elimination of DFC in these experiments. This fact has been previously demonstrated in several native fungal species from polluted environments, in which the xenobiotic detoxification system was responsible for the degradation of DFC and polycyclic aromatic hydrocarbons (PAHs) [66,71,72].

The pH showed a slight increase and the biomass did not show significant changes during the experiments. However, a trend was observed between the elimination of DFC and the nutrient concentrations; this would indicate that a possible co-metabolic process occurs between the degradation and the amount of glucose (Figure 2). This is consistent with the degradation results of DFC with the fungus *Penicillium oxalicum* [31]. Finally, the abiotic control of *S. monosporum* (Figure 2b) and *T. verruculosus* (Figure 2d) exhibited lightly reduced elimination (<30%), and a bio-absorption process or adhesion capacity could be involved.

These species belong mostly to the Ascomycota or Mucoromycota phyla that have been reported before in wastewater treatment occurrence [73]. Many other ascomycete species have shown degradation of EPs, such as of *P. oxalicum,* which have been studied under several sources including real hospital wastewater [68]. This fungus, commonly found in soil and water, has been used under laboratory conditions to degrade polythene [74], 4,6-trinitrotoluene [75], and chlorinated PAHs [76]. However, some of the species reported here have not been reported before in DFC removal, but some related strains (*Aspergillus terreus* CCS2B, *Talaromyces spectabilis CCS12*) degraded mixtures of PAHs [51] and pyrene oxidation by *Syncephalastrum racemosum* [76].

### 3.5. Bisphenol A Degradation Experiments

The degradation efficiency of BPA in the isolated fungi was also evaluated. We selected the following 3 of the 17 isolated fungi for their ability to transform BPA (Table 1): *Talaromyces gossypii, Syncephalastrum monosporum*, and *Talaromyces verruculosus*. 

**Figure 3:**
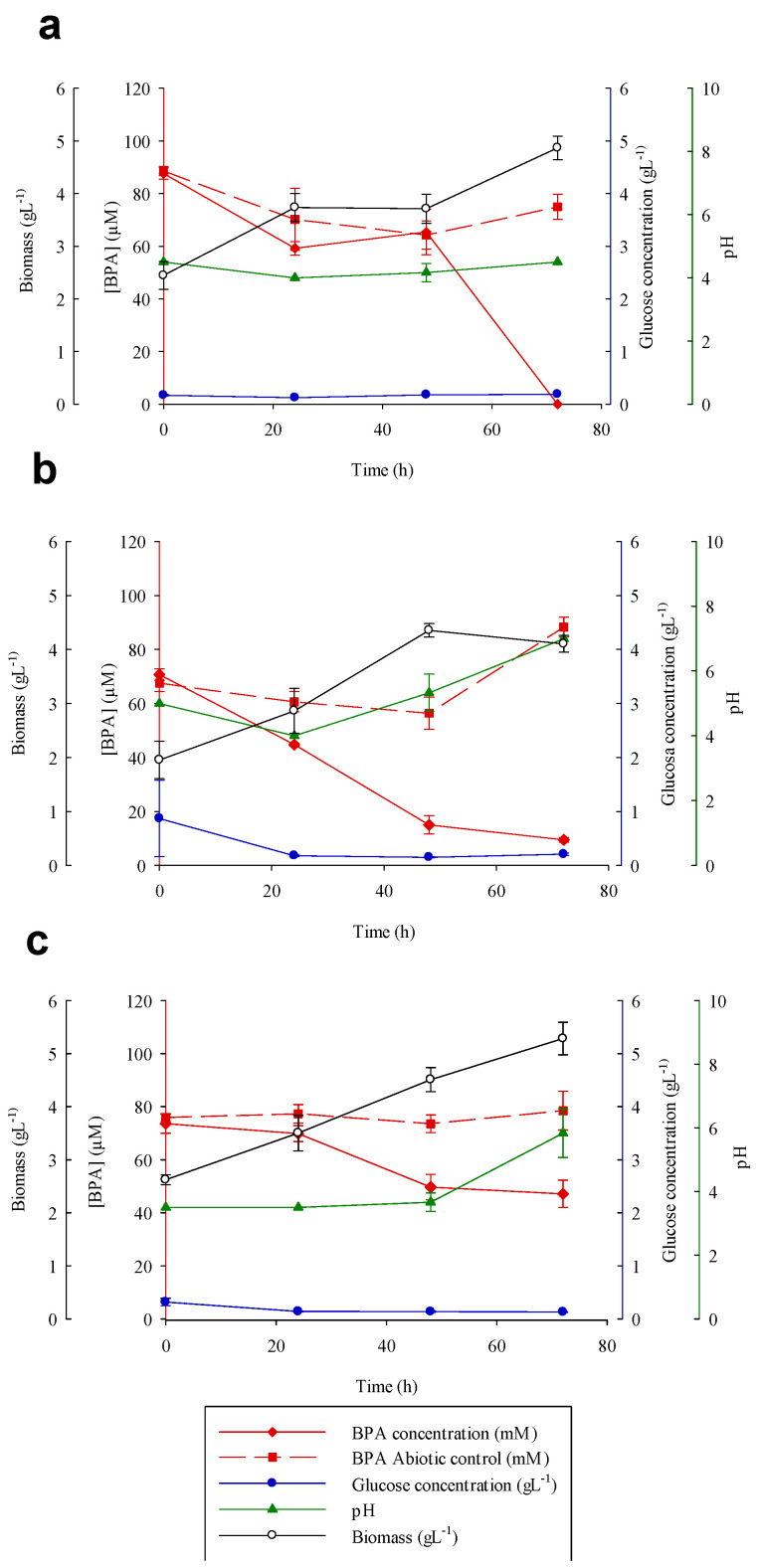
Comparison of bisphenol A elimination by *Syncephalastrum monosporum* (**a**), *Talaromyces gossypii* (**b**), and *Talaromyces verruculosus* (**c**). Bisphenol A concentration (red diamond), glucose concentration (blue circles), pH (green triangle), biomass (white circles), and the bisphenol abiotic control (red square) were measured. Error bars represent the standard deviation of three independent replicates.

Figure 3 illustrates that after 48 h of treatment with *S. monosporum* or *T. gossypii,* more than 80% of the BPA was removed, and complete degradation (100%) occurred in *S. monosporum* at 72 h. Moreover*, T. verruculosum* gave the lowest BPA removal (36%) after 72 h of treatment.

MnP activity was observed in the three species (i.e., *T. gossypii, S. monosporum,* and *T. verruculosus*) that seemed to be induced by BPA, since in the presence of DFC it was not secreted. MnP was not statistically different among the fungi. However, MnP activity reached higher values in *T. verruculosus* (11.11 ± 1.44 U mL^-1^; 24 h) and in *S. racemosum* (7.02 ± 0.47 U mL^-1^; 48 h) (data not shown). Thus, MnP may have a role in the removal of BPA. However, we cannot discard the participation or contribution of the xenobiotic detoxification systems in this process as we stated for DFC degradation. 

Several studies have demonstrated the participation of oxidase enzymes in the biodegradation/biotransformation of BPA, in particular fungi that produce laccases [77,78]. Daâssi et al. [79] reported the efficiency of laccase from *Coriolopsis gallica*, *Bjerkandera adusta,* and *T. versicolor* in the biodegradation of BPA; *C. gallica* had the highest BPA removal rate (91%). Shin et al. [80] reported that the degradation of BPA by the fungus *Irpex lacteus* could be mediated by laccase (0.5 U mL^-1^) and MnP (7.5 U mL^-1^). Similarly, the basidiomycete *Pleurotus ostreatus* produces MnP in the presence of BPA and seems to be involved in the transformation of BPA to phenol among other phenolic compounds [81].

The pH increased slightly in the BPA degradation systems with *T. gossypii* and *T. verruculosus*. In the case of the fungus *S. monosporum,* the pH did not change during the experiment. In all of the analyzed fungal strains, no correlations were observed in the concentration of nutrients and BPA degradation (Figure 3). 

Bio-absorption was observed in the present study, in which the BPA concentration decreased to 9.1 ± 4.8%, 20.3 ± 7.7%, and 40.8 ± 6.4% at the end of the experiment in the tests with the abiotic control of fungal mycelium (*T. gossypii, S. monosporum, T. verruculosus*, respectively). This shows the absorption and adhesion capacities of BPA to the polysaccharides of the fungal cell walls.

Therefore, during this study, *S. racemosum* showed the highest adhesion levels (40.8 ± 6.4%). This phenomenon was previously observed in other studies with ascomycetes fungi, such as *Thielavia arenaria* HJ22, which produced a decrease in the concentration of BPA in the culture medium to 70 μM on heat-inactivated mycelium [82]. 

## 4. Conclusions

The results of this study revealed that sewage sludge could represent an interesting source for the isolation of different groups of fungi for further applications in degradation. Biological fungal systems can be an effective strategy for the development of different applications in WWTPs, conferring economic advantages in a standard period of time. Further experiments are needed in order to decipher the role of bacteria in these samples and the possibility to perform microbial consortia.

## Figures and Tables

**Table 1:** Diclofenac and bisphenol A consumption rates and GenBank accession numbers of the fungal strains included in this study.

Accession Number	BLAST Percent of Identity	Phylum	Isolated Fungi Strain	Total DFC Consumption	Total BPA Consumption
In correction	99.34	Ascomycota	*Talaromyces gossypii*	84.6%	86.6%
MW931877	96.30	Zygomycota	*Syncephalastrum monosporum*	82%	100%
In correction	99.16	Ascomycota	*Aspergillus tabacinus*	76%	-
MW931860	100	Ascomycota	*Talaromyces verruculosus*	37%	36%
MT792070	100	Ascomycota	*Aspergillus terreus*	49.7%	-
MW931880	100	Ascomycota	*Aspergillus cejpii*	14.6%	-
MW931859	100	Ascomycota	*Galactomyces candidum*	-	-
MW931874	100	Ascomycota	*Galactomyces geotrichum*	-	-
MT792001	100	Ascomycota	*Saccharomycetales sp.*	-	-
MW931860	100	Ascomycota	*Aspergillus cremeus*	-	-
MT792005	100	Ascomycota	*Byssochlamys nivea*	-	-
MT787659	100	Ascomycota	*Trichoderma asperellum*	-	-
MT792081	95.34	Ascomycota	*Scedosporium aurantiacum*	-	-
MT792234	100	Ascomycota	*Sporothrix mexicana*	-	-
MT792235	100	Ascomycota	*Aspergillus sydowii*	-	-
MT787565	100	Mucoromyceta	*Mucor circinelloides*	-	-
MW931863	99.82	Ascomycota	*Talaromyces pinophilus*	-	-

## Data Availability

The ITS sequences were deposited in the GenBank database (https://www.ncbi.nlm.nih.gov) under the access number cited in Table 1.

## Supplementary Materials

The following are available online at https://www.mdpi.com/article/10.3390/toxics9060115/s1, Table S1: the composition of the bacterial community (genus) detected at <0.5% abundance in sewage sludge samples.

## References

[1] Geissen V., Mol H., Klumpp E., Umlauf G., Nadal M., van der Ploeg M., van de Zee S.E.A.T.M., Ritsema C.J. (2015). Emerging pollutants in the environment: A challenge for water resource management. Int. Soil Water Conserv. Res..

[2] Petrie B., Barden R., Kasprzyk-Hordern B. (2015). A review on emerging contaminants in wastewaters and the environment: Current knowledge, understudied areas and recommendations for future monitoring. Water Res..

[3] Verlicchi P., Galletti A., Petrovic M., Barceló D. (2010). Hospital effluents as a source of emerging pollutants: An overview of micropollutants and sustainable treatment options. J. Hydrol..

[4] Al Aukidy M., Verlicchi P., Jelic A., Petrovic M., Barcelò D. (2012). Monitoring release of pharmaceutical compounds: Occurrence and environmental risk assessment of two WWTP effluents and their receiving bodies in the Po Valley, Italy. Sci. Total Environ..

[5] Alexander J.T., Hai F.I., Al-aboud T.M. (2012). Chemical coagulation-based processes for trace organic contaminant removal: Current state and future potential. J. Environ. Manag..

[6] Verlicchi P., Al Aukidy M., Zambello E. (2012). Occurrence of pharmaceutical compounds in urban wastewater: Removal, mass load and environmental risk after a secondary treatment—A review. Sci. Total Environ..

[7] Verlicchi P., Zambello E. (2015). Pharmaceuticals and personal care products in untreated and treated sewage sludge: Occurrence and environmental risk in the case of application on soil—A critical review. Sci. Total Environ..

[8] Pal A., He Y., Jekel M., Reinhard M., Gin K.Y.-H. (2014). Emerging contaminants of public health significance as water quality indicator compounds in the urban water cycle. Environ. Int..

[9] Tran N.H., Reinhard M., Gin K.Y.-H. (2018). Occurrence and fate of emerging contaminants in municipal wastewater treatment plants from different geographical regions—A review. Water Res..

[10] Grelska A., Noszczyńska M. (2020). White rot fungi can be a promising tool for removal of bisphenol A, bisphenol S, and nonylphenol from wastewater. Environ. Sci. Pollut. Res..

[11] Vieno N., Sillanpää M. (2014). Fate of diclofenac in municipal wastewater treatment plant—A review. Environ. Int..

[12] Schug T.T., Birnbaum L.S., Snedeker S.M. (2014). Human Health Effects of Bisphenol A. Toxicants in Food Packaging and Household Plastics: Exposure and Health Risks to Consumers.

[13] Larsen G.D. (2015). Transgenerational effects of BPA. Lab. Animal.

[14] Calvo-Flores F.G., Isac-García J., Dobado J.A. (2017). Emerging Pollutants: Origin, Structure and Properties. Emerging Pollutants: Origin, Structure and Properties.

[15] Bilal M., Iqbal H.M.N., Barceló D. (2019). Mitigation of bisphenol A using an array of laccase-based robust bio-catalytic cues—A review. Sci. Total Environ..

[16] Quintana J.B., Weiss S., Reemtsma T. (2005). Pathways and metabolites of microbial degradation of selected acidic pharmaceutical and their occurrence in municipal wastewater treated by a membrane bioreactor. Water Res..

[17] Yu J.T., Bouwer E.J., Coelhan M. (2006). Occurrence and biodegradability studies of selected pharmaceuticals and personal care products in sewage effluent. Agric. Water Manag..

[18] Li X., Zhou M., Pan Y. (2018). Degradation of diclofenac by H_2_O_2_ activated with pre-magnetization Fe^0^: Influencing factors and degradation pathways. Chemosphere.

[19] Ho-Young J., Jin-Kyu K., Seung-Chan L., Jeong-Ann P., Song-Bae K. (2021). Analysis of diclofenac removal by metal-organic framework MIL-100(Fe) using multi-parameter experiments and artificial neural network modeling. J. Taiwan Inst. Chem. Eng..

[20] Jae-Hun C., Jin-Kyu K., Seong-Jik P., Chang-Gu L. (2021). Bisphenol A degradation using waste antivirus copper film with enhanced sono-Fenton-like catalytic oxidation. Chemosphere.

[21] Angosto J.M., Roca M.J., Fernández-López J.A. (2020). Removal of Diclofenac in Wastewater Using Biosorption and Advanced Oxidation Techniques: Comparative Results. Water.

[22] Wang J., Wang S. (2016). Removal of pharmaceuticals and personal care products (PPCPs) from wastewater: A review. J. Environ. Manag..

[23] Sun C., Li W., Chen Z., Qin W., Wen X. (2019). Responses of antibiotics, antibiotic resistance genes, and mobile genetic elements in sewage sludge to thermal hydrolysis pre-treatment and various anaerobic digestion conditions. Environ. Int..

[24] Bhat S.A., Cui G., Li W., Wei Y., Li F. (2020). Effect of heavy metals on the performance and bacterial profiles of activated sludge in a semi-continuous reactor. Chemosphere.

[25] Zhang J., Zhao R., Cao L., Lei Y., Liu J., Feng J., Fu W., Li X., Li B. (2020). High-efficiency biodegradation of chloramphenicol by enriched bacterial consortia: Kinetics study and bacterial community characterization. J. Hazard. Mater..

[26] Sankaran S., Khanal S.K., Jasti N., Jin B., Pometto A.L., Van Leeuwen J.H. (2010). Use of Filamentous Fungi for Wastewater Treatment and Production of High Value Fungal Byproducts: A Review. Crit. Rev. Environ. Sci. Technol..

[27] Mir-Tutusaus J.A., Baccar R., Caminal G., Sarrà M. (2018). Can white-rot fungi be a real wastewater treatment alternative for organic micropollutants removal? A review. Water Res..

[28] Silva A., Delerue-Matos C., Figueiredo S.A., Freitas O.M. (2019). The Use of Algae and Fungi for Removal of Pharmaceuticals by Bioremediation and Biosorption Processes: A Review. Water.

[29] More T.T., Yan S., Tyagi R.D., Surampalli R.Y. (2010). Potential use of filamentous fungi for wastewater sludge treatment. Bioresour. Technol..

[30] Cruz-Morató C., Lucas D., Llorca M., Rodriguez-Mozaz S., Gorga M., Petrovic M., Barceló D., Vicent T., Sarrà M. (2014). Marco-Urrea, E. Hospital wastewater treatment by fungal bioreactor: Removal efficiency for pharmaceuticals and endocrine disruptor compounds. Sci. Total Environ..

[31] Olicón-Hernández D.R., Camacho-Morales R.L., Pozo C., González-López J., Aranda E. (2019). Evaluation of diclofenac biodegradation by the ascomycete fungus *Penicillium oxalicum* at flask and bench bioreactor scales. Sci. Total Environ..

[32] Chai W., Handa Y., Suzuki M., Saito M., Kato N., Horiuchi C.A. (2005). Biodegradation of bisphenol a by fungi. Appl. Biochem. Biotechnol..

[33] Marco-Urrea E., García-Romera I., Aranda E. (2015). Potential of non-ligninolytic fungi in bioremediation of chlorinated and polycyclic aromatic hydrocarbons. New Biotech..

[34] Aracagök Y., Goker H., Cihangir N. (2018). Biodegradation of diclofenac with fungal strains. Arch. Environ. Prot..

[35] D’Annibale A., Rosetto F., Leonardi V., Federici F., Petruccioli M. (2006). Role of Autochthonous Filamentous Fungi in Bioremediation of a Soil Historically Contaminated with Aromatic Hydrocarbons. Appl. Environ. Microbiol..

[36] Mishra A., Malik A. (2014). Novel fungal consortium for bioremediation of metals and dyes from mixed waste stream. Bioresour. Technol..

[37] Sharma S., Malaviya P. (2016). Bioremediation of tannery wastewater by chromium resistant novel fungal consortium. Ecol. Eng..

[38] Waksman S.A. (1922). A Method for Counting the Number of Fungi in the Soil. J. Bacteriol..

[39] Vesty A., Biswas K., Taylor M.W., Gear K., Douglas R.G. (2017). Evaluating the Impact of DNA Extraction Method on the Representation of Human Oral Bacterial and Fungal Communities. PLoS ONE.

[40] Thompson L.R., Sanders J.G., McDonald D., Amir A., Ladau J., Locey K.J., Prill R.J., Tripathi A., Gibbons S.M., Ackermann G. (2017). A communal catalogue reveals Earth’s multiscale microbial diversity. Nature.

[41] Caporaso J.G., Lauber C.L., Walters W.A., Berg-Lyons D., Lozupone C.A., Turnbaugh P.J., Fierer N., Knight R. (2011). Global patterns of 16S rRNA diversity at a depth of millions of sequences per sample. Proc. Natl. Acad. Sci. USA.

[42] White T.J., Bruns T., Lee S., Taylor J., Innis M.A., Gelfand D.H., Sninsky J.J., White T.J. (1990). Amplification and Direct Sequencing of Fungal Ribosomal RNA Genes for Phylogenetics. PCR Protocols.

[43] Caporaso J.G., Lauber C.L., Walters W.A., Berg-Lyons D., Huntley J., Fierer N., Owens S.M., Betley J., Fraser L., Bauer M. (2012). Ultra-high-throughput microbial community analysis on the Illumina HiSeq and MiSeq platforms. ISME J..

[44] Bolyen E., Rideout J.R., Dillon M.R., Bokulich N.A., Abnet C.C., Al-Ghalith G.A., Alexander H., Alm E.J., Arumugam M., Asnicar F. (2019). Reproducible, interactive, scalable and extensible microbiome data science using QIIME 2. Nat. Biotechnol..

[45] Callahan B.J., McMurdie P.J., Rosen M.J., Han A.W., Johnson A.J.A., Holmes S.P. (2016). DADA2: High-resolution sample inference from Illumina amplicon data. Nat. Methods.

[46] Kirk T.K., Schultz E., Connors W.J., Lorenz L.F., Zeikus J.G. (1978). Influence of culture parameters on lignin metabolism by *Phanerochaete chrysosporium*. Arch. Microbiol..

[47] Miller G.L. (1959). Use of Dinitrosalicylic Acid Reagent for Determination of Reducing Sugar. Anal. Chem..

[48] Eggert C., Temp U., Dean J.F.D., Eriksson K.-E.L. (1995). Laccase-mediated formation of the phenoxazinone derivative, cinnabarinic acid. FEBS Lett..

[49] Wariishi H., Valli K., Gold M.H. (1992). Manganese(II) oxidation by manganese peroxidase from the basidiomycete *Phanerochaete chrysosporium*. Kinetic mechanism and role of chelators. J. Biol. Chem..

[50] Ullrich R., Nüske J., Scheibner K., Spantzel J., Hofrichter M. (2004). Novel Haloperoxidase from the Agaric Basidiomycete *Agrocybe aegerita* Oxidizes Aryl Alcohols and Aldehydes. Appl. Environ. Microbiol..

[51] Reyes-César A., Absalón Á.E., Fernández F.J., González J.M., Cortés-Espinosa D.V. (2014). Biodegradation of a mixture of PAHs by non-ligninolytic fungal strains isolated from crude oil-contaminated soil. World J. Microbiol. Biotechnol..

[52] DeSantis T.Z., Hugenholtz P., Larsen N., Rojas M., Brodie E.L., Keller K., Huber T., Dalevi D., Hu P., Andersen G.L. (2006). Greengenes, a chimera-checked 16S rRNA gene database and workbench compatible with ARB. Appl. Environ. Microbiol..

[53] Pérez-Díaz M.I., Zárate-Segura P., Bermeo-Fernández L.A., Nirmalkar K., Bastida-González F., García-Mena J., Jan-Roblero J., Guerrero-Barajas C. (2020). Bacterial consortium from hydrothermal vent sediments presents electrogenic activity achieved under sulfate reducing conditions in a microbial fuel cell. J. Environ. Health Sci. Eng..

[54] Costa J.M., Rodriguez R.P., Sancinetti G.P. (2017). Removal sulfate and metals Fe^+2^, Cu^+2^, and Zn^+2^ from acid mine drainage in an anaerobic sequential batch reactor. J. Environ. Chem. Eng..

[55] Martins M., Faleiro M.L., Barros R.J., Veríssimo A.R., Barreiros M.A., Costa M.C. (2009). Characterization and activity studies of highly heavy metal resistant sulphate-reducing bacteria to be used in acid mine drainage decontamination. J. Hazard. Mater..

[56] Militon C., Hamdi O., Michotey V., Fardeau M.L., Ollivier B., Bouallagui H., Hamdi M., Bonin P. (2015). Ecological significance of Synergistetes in the biological treatment of tuna cooking wastewater by an anaerobic sequencing batch reactor. Environ. Sci. Pollut. Res. Int..

[57] Elshahed M.S., Bhupathiraju V.K., Wofford N.Q., Nanny M.A., McInerney M.J. (2001). Metabolism of benzoate, cyclohex-1-ene carboxylate, and cyclohexane carboxylate by “*Syntrophus aciditrophicus*” strain SB in syntrophic association with H(2)-using microorganisms. Appl. Environ. Microbiol..

[58] Grimm F., Franz B., Dahl C. (2011). Regulation of dissimilatory sulfur oxidation in the purple sulfur bacterium allochromatium vinosum. Front. Microbiol..

[59] Kragelund C., Thomsen T.R., Mielczarek A.T., Nielsen P.H. (2011). Eikelboom’s morphotype 0803 in activated sludge belongs to the genus *Caldilinea* in the phylum *Chloroflexi*. FEMS Microbiol. Ecol..

[60] Yoon D.N., Park S.J., Kim S.J., Jeon C.O., Chae J.C., Rhee S.K. (2010). Isolation, characterization, and abundance of filamentous members of *Caldilineae* in activated sludge. J. Microbiol..

[61] Ju F., Guo F., Ye L., Xia Y., Zhang T. (2014). Metagenomic analysis on seasonal microbial variations of activated sludge from a full-scale wastewater treatment plant over 4 years. Environ. Microbiol. Rep..

[62] Bogan B.W., Lahner L.M., Sullivan W.R., Paterek J.R. (2003). Degradation of straight-chain aliphatic and high-molecular-weight polycyclic aromatic hydrocarbons by a strain of *Mycobacterium austroafricanum*. J. Appl. Microbiol..

[63] Dudhagara D., Dave B., Ribón W. (2018). Mycobacterium as Polycyclic Aromatic Hydrocarbons (PAHs) Degrader. Mycobacterium—Research and Development.

[64] Rojo F., Martínez J.L., Goldfine H. (2020). Hydrocarbon Degraders as Pathogens. Health Consequences of Microbial Interactions with Hydrocarbons, Oils, and Lipids.

[65] Nilsson R.H., Larsson K.-H., Taylor A.F.S., Bengtsson-Palme J., Jeppesen T.S., Schigel D., Kennedy P., Picard K., Glöckner F.O., Tedersoo L. (2018). The UNITE database for molecular identification of fungi: Handling dark taxa and parallel taxonomic classifications. Nucleic Acids Res..

[66] Aranda E., Godoy P., Reina R., Badia-Fabregat M., Rosell M., Marco-Urrea E., García-Romera I. (2017). Isolation of Ascomycota fungi with capability to transform PAHs: Insights into the biodegradation mechanisms of *Penicillium oxalicum*. Int. Biodeterior. Biodegrad..

[67] Godoy P., Reina R., Calderón A., Wittich R.-M., García-Romera I., Aranda E. (2016). Exploring the potential of fungi isolated from PAH-polluted soil as a source of xenobiotics-degrading fungi. Environ. Sci. Pollut. Res..

[68] Olicón-Hernández D.R., Gómez-Silván C., Pozo C., Andersen G.L., González-Lopez J., Aranda E. (2021). *Penicillium oxalicum* XD-3.1 removes pharmaceutical compounds from hospital wastewater and outcompetes native bacterial and fungal communities in fluidised batch bioreactors. Int. Biodeterior. Biodegrad..

[69] Assress H.A., Selvarajan R., Nyoni H., Ntushelo K., Mamba B.B., Msagati T.A.M. (2019). Diversity, Co-occurrence and Implications of Fungal Communities in Wastewater Treatment Plants. Sci. Rep..

[70] Maza-Márquez P., Aranda E., González-López J., Rodelas B., Biassoni R., Raso A. (2020). Evaluation of the Abundance of Fungi in Wastewater Treatment Plants Using Quantitative PCR (qPCR). Quantitative Real-Time PCR: Methods and Protocols.

[71] Hata T., Kawai S., Okamura H., Nishida T. (2010). Removal of diclofenac and mefenamic acid by the white rot fungus *Phanerochaete sordida* YK-624 and identification of their metabolites after fungal transformation. Biodegradation.

[72] Lucero Camacho-Morales R., García-Fontana C., Fernández-Irigoyen J., Santamaría E., González-López J., Manzanera M., Aranda E. (2018). Anthracene drives sub-cellular proteome-wide alterations in the degradative system of *Penicillium oxalicum*. Ecotoxicol. Environ. Safe..

[73] Liu L., Wang S., Chen J. (2020). Hysteretic response of Microbial Eukaryotic Communities to Gradually Decreased Nutrient Concentrations in Eutrophic Water. Microb. Ecol..

[74] Sangale M.K., Shahnawaz M., Ade A.B. (2019). Potential of fungi isolated from the dumping sites mangrove rhizosphere soil to degrade polythene. Sci. Rep..

[75] Anasonye F., Winquist E., Räsänen M., Kontro J., Björklöf K., Vasilyeva G., Jørgensen K.S., Steffen K.T., Tuomela M. (2015). Bioremediation of TNT contaminated soil with fungi under laboratory and pilot scale conditions. Int. Biodeterior. Biodegrad..

[76] Launen L., Pinto L., Wiebe C., Kiehlmann E., Moore M. (1995). The oxidation of pyrene and benzo[a]pyrene by nonbasidiomycete soil fungi. Can. J. Microbiol..

[77] Dalecka B., Oskarsson C., Juhna T., Kuttava Rajarao G. (2020). Isolation of Fungal Strains from Municipal Wastewater for the Removal of Pharmaceutical Substances. Water.

[78] Martínková L., Kotik M., Marková E., Homolka L. (2016). Biodegradation of phenolic compounds by Basidiomycota and its phenol oxidases: A review. Chemosphere.

[79] Daâssi D., Prieto A., Zouari-Mechichi H., Martínez M.J., Nasri M., Mechichi T. (2016). Degradation of bisphenol A by different fungal laccases and identification of its degradation products. Int. Biodeterior. Biodegrad..

[80] Shin E.H., Choi H.T., Song H.G. (2007). Biodegradation of endocrine-disrupting bisphenol A by white rot fungus *Irpex lacteus*. J. Microbiol. Biotechnol..

[81] Hirano T., Honda Y., Watanabe T., Kuwahara M. (2000). Degradation of Bisphenol A by the Lignin-Degrading Enzyme, Manganese Peroxidase, Produced by the White-rot Basidiomycete, *Pleurotus ostreatus*. Biosci. Biotechnol. Biochem..

[82] Mtibaà R., Olicón-Hernández D.R., Pozo C., Nasri M., Mechichi T., González J., Aranda E. (2018). Degradation of bisphenol A and acute toxicity reduction by different thermo-tolerant ascomycete strains isolated from arid soils. Ecotoxicol. Environ. Safe.

