# Supplementary material for: Evaluation of the Potential of Sewage Sludge Mycobiome to Degrade High Diclofenac and Bisphenol-A Concentrations
Source: Toxics. 2021 May 23;9(6):115. doi: 10.3390/toxics9060115 (PMC8224792; doi:10.3390/toxics9060115)
Supplement: Supplementary file 1 [file toxics-09-00115-s001.zip › toxics-1221215-supplementary.pdf]

Toxics **2021**, *9*, **115**; doi:10.3390/toxics9060115

Supplementary Materials: Evaluation of the Potential of Sewage Sludge Mycobiome to Degrade High Diclofenac and Bisphenol-A Concentrations

Ulises Conejo-Saucedo, Alejandro Ledezma-Villanueva, Gabriela Ángeles de Paz, Mario Herrero-Cervera, Concepción Calvo and Elisabet Aranda

Table S1. The composition of the bacterial community (genus) detected at <0.5% abundance in sewage sludge samples.

Family	Genus
Rhodobacteraceae	*Rhodobacter*
	Paracoccus
Clostridiaceae	*Clostridium*
	Proteiniclasticum
Methanobacteriaceae	*Methanobrevibacter*
	Methanosphaera
	Methanobacterium
Geobacteraceae	*Geobacter*
Peptococcaceae	*Pelotomaculum*
Sphingomonadaceae	*Novosphingobium*
	Sphingopyxis
Rhodocyclaceae	*Dechloromonas*
	Azospira
	Propionivibrio
	Sulfuritalea
Dietziaceae	*Dietzia*
Intrasporangiaceae	*Phycicoccus*
Beijerinckiaceae	*Chelatococcus*
Spirochaetaceae	*Treponema*
Comamonadaceae	*Acidovorax*
	Comamonas
	Hydrogenophaga
	Rhodoferax
	Rubrivivax
Planctomycetaceae	*Planctomyces*
Hyphomicrobiaceae	*Hyphomicrobium*
	Devosia
	Rhodoplanes
Nocardioidaceae	*Nocardioides*
Microbacteriaceae	*Leucobacter*
	Cryocola
Anaerolinaceae	*Anaerolinea*
	Longilinea
Syntrophaceae	*Desulfomonile*
	Syntrophobacter
Caulobacteraceae	*Brevundimonas*
	Phenylobacterium
Enterococcaceae	*Enterococcus*
Eubacteriaceae	*Acetobacterium*

	Pseudoramibacter Eubacterium
	Gracilibacter
Methanospirillaceae	*Methanospirillum*
Thermotogaceae	*Kosmotoga*
Bacteroidaceae	*Bacteroides*
Porphyromonadaceae	*Macellibacteroides*
	Paludibacter
	Petrimonas
Xanthomonadaceae	*Dokdonella*
	Luteimonas
	Pseudoxanthomonas
	Stenotrophomonas
	Thermomonas
Isosphaeraceae	*Nostocoida*
Mogibacteriaceae	*Anaerovorax*
Phyllobacteriaceae	*Aminobacter*
	Chelativorans
	Mesorhizobium
Acetobacteraceae	*Roseomonas*
Desulfovibrionaceae	*Desulfovibrio*
Turicibacteraceae	*Turicibacter*
Cloacamonaceae	*Candidatus Cloacamonas*
Coxiellaceae	*Aquicella*
	Legionella
	Tatlockia
Rhodospirillaceae	*Defluviicoccus*
	Dongia
	Reyranella
Gordoniaceae	*Gordonia*
Corynebacteriaceae	*Corynebacterium*
Bdellovibrionaceae	*Bdellovibrio*
Moraxellaceae	*Acinetobacter*
Lachnospiraceae	*Anaerostipes*
	Blautia
	Butyrivibrio
	Coprococcus
	Roseburia
Ruminococcaceae	*Faecalibacterium*
	Gemmiger
	Oscillospira
	Ruminococcus
Chthoniobacteraceae	*Candidatus Xiphinematobacter*
Enterococcaceae	*Enterococcus*
	Lactococcus
	Streptococcus
Halomonadaceae	*Halomonas*
Actinomycetaceae	*Actinomyces*
Methanoregulaceae	*Methanolinea*
Tissierellaceae	*Gallicola*
	Sedimentibacter
	Tissierella_Soehngenia

Neisseriaceae	*Uruburuella*
Bifidobacteriaceae	*Bifidobacterium*
Gemmataceae	*Gemmata*
Rhizobiaceae	*Kaistia*
	Shinella
	Sinorhizobium
Methanomassiliicoccaceae	*Methanomassiliicoccus*
Veillonellaceae	*Acidaminococcus*
	Anaeromusa
	Anaerovibrio
	Phascolarctobacterium
	Succinispira
	Veillonella
Chitinophagaceae	*Niabella*
Desulfobulbaceae	*Desulfobulbus*
Coriobacteriaceae	*Collinsella*
Dehalobacteriaceae	*Dehalobacterium*
Prevotellaceae	*Prevotella*
Xanthobacteraceae	*Blastochloris*
	Xanthobacter
Parachlamydiaceae	*Candidatus Protochlamydia*
Leptotrichiaceae	*Sebaldella*
Burkholderiaceae	*Burkholderia*
Brucellaceae	*Ochrobactrum*
Weeksellaceae	*Cloacibacterium*
Criblamydiaceae	*Estrella*
Flavobacteriaceae	*Flavobacterium*
Pirellulaceae	*Pirellula*
Beutenbergiaceae	*Salana*
Rikenellaceae	*Alistipes*
Alcaligenaceae	*Bordetella*
Microthrixaceae	*Candidatus Microthrix*
Pseudomonadaceae	*Pseudomonas*
Odoribacteraceae	*Butyricimonas*
Verrucomicrobiaceae	*Luteolibacter*
	Prosthecobacter
Erysipelotrichaceae	*Eubacterium*
Nocardiaceae	*Rhodococcus*
Geodermatophilaceae	*Blastococcus*
